# Serum extracellular matrix biomarkers in rheumatoid arthritis, psoriatic arthritis and psoriasis and their association with hand function

**DOI:** 10.1038/s41598-025-98395-0

**Published:** 2025-04-21

**Authors:** Helena Port, Birte Coppers, Sonja Tragl, Eva Manger, Lisa M. Niemiec, Sara Bayat, David Simon, Filippo Fagni, Giulia Corte, Anne-Christine Bay-Jensen, Koray Tascilar, Axel J. Hueber, Katja G. Schmidt, Verena Schönau, Michael Sticherling, Simon Heinrich, Sigrid Leyendecker, Daniela Bohr, Georg Schett, Arnd Kleyer, Signe Holm Nielsen, Anna-Maria Liphardt

**Affiliations:** 1 University of Copenhagen, Copenhagen, Denmark; 2 Nordic Bioscience, Herlev, Denmark; 3 Department of Internal Medicine 3 - Rheumatology and Immunology, Friedrich-Alexander-Universität Erlangen-Nürnberg and Universitätsklinikum Erlangen, Erlangen, Germany; 4 Deutsches Zentrum Immuntherapie, Friedrich-Alexander-Universität Erlangen-Nürnberg and Universitätsklinikum Erlangen, Erlangen, Germany; 5 Department of Internal Medicine, Clinical Division of Internal Medicine II, Medical University Innsbruck/Tirol Kliniken GmbH, Innsbruck, Austria; 6 Elisabeth-Krankenhaus Kassel, Kassel, Germany; 7 Charité Universitätsmedizin Berlin, Med. Klinik mit Schwerpunkt Rheumatologie und Klinische Immunologie Berlin, Berlin, Germany; 8 Division of Rheumatology, Klinikum Nürnberg, Paracelsus Medical University, Nürnberg, Germany; 9 Departement of Dermatology, Friedrich-Alexander-Universität Erlangen-Nürnberg and Universitätsklinikum Erlangen, Erlangen, Germany; 10 Institute of Applied Dynamics, Friedrich-Alexander-Universität Erlangen-Nürnberg, Erlangen, Germany

**Keywords:** Extracellular matrix, Biochemical markers, Rheumatoid arthritis, Psoriatic arthritis, Psoriasis, Hand function, Post-translational modifications, Immunology, Molecular biology, Rheumatology, Rheumatoid arthritis, Biomarkers, Diagnostic markers, Skin diseases

## Abstract

Inflammatory arthritis, including rheumatoid arthritis and psoriatic arthritis, is characterized by physical function impairment. This becomes apparent even before arthritis onset, as in psoriasis (PsO). Chronic inflammation triggers an accelerated remodeling of the extracellular matrix (ECM), resulting in released ECM fragments detectable in blood. We aimed to investigate levels of blood-based ECM biomarkers in patients with RA, PsA, PsO, and healthy controls and to explore the association of ECM biomarkers with hand function impairments. Patients with RA (n = 85), PsA (n = 115), PsO (n = 102) and controls (n = 110) were included in this cross-sectional study. ECM catabolic (C1M, C2M, C3M, C4M, PRO-C4, C6M, ARG), formation (PRO-C1, PRO-C3, PRO-C6) and inflammation biomarkers (VICM) were measured in serum from all patients. Objective hand function (fine motor skills (Moberg-Picking-Up Test), isometric grip strength (dynamometer) and patient-perceived hand function (Michigan Hand Questionnaire (MHQ)) were assessed. Patients with RA and PsA received treatment with disease-modifying anti-rheumatic drugs. VICM levels were higher in RA, PsA, and PsO than in controls (p < 0.0001). PsA and PsO showed higher C4M levels compared to controls (p < 0.0001, p < 0.0001), while C6M was lower in patients with RA, PsA and PsO than in controls (p < 0.0001, p < 0.001, p < 0.01). PsO presented with higher levels of C1M compared to controls and to RA (p < 0.001 and p < 0.0001). PRO-C6 correlated negatively with MHQ (*ρ* = -0.39, p < 0.01) and grip strength *(ρ* = -0.31, p < 0.05) in PsO, while only weak correlations were observed between biomarkers and hand function scores for RA and PsA patients (all *ρ* <  ± 0.2–0.3). Patients with RA, PsA, and PsO showed significant alterations in ECM remodeling biomarkers. Especially PsA and PsO had higher levels of inflammatory biomarkers compared to RA and controls, likely due to modulation by treatment. Predominantly in PsO, ECM formation biomarkers were associated with hand function impairments.

## Introduction

Rheumatoid arthritis (RA), psoriatic arthritis (PsA), and psoriasis (PsO) are chronic inflammatory disorders that primarily affect the musculoskeletal system and the skin. RA is characterized by joint inflammation and cartilage degradation, leading to pain, swelling, and stiffness^1^. PsO is a chronic auto-immune skin disease with erythematous and scaly patches in the skin, resulting from abnormal proliferation and differentiation of epidermal keratinocytes. Because up to 30% of patients with PsO progress to PsA (characterized by inflammatory arthritis, enthesitis, dactylitis and spondylitis^2^) PsO patients are considered an at-risk population to develop PsA. RA, PsA and PsO share several clinical features and have altered tissue turnover of the joints and skin, respectively^3,4^. To avoid structural damage of the musculoskeletal system, timely treatment at disease onset or change of treatment with flare is the goal, and sensitive, pragmatic biomarkers for the detection of disease onset or flare are needed.

The extracellular matrix (ECM) consists of proteins, including collagen and proteoglycans, which provide support and elasticity to various tissues of joints and skin^5^. Type I and III collagens are the most abundant proteins in soft tissues, providing structural support, while type II collagen and aggrecan are key proteins in articular cartilage, maintaining tissue integrity^5,6^. Type IV collagen forms the basement membrane, acting as a barrier between tissue compartments^5,7^, and Type VI collagen contributes to tissue organization and stability of connective tissues such as the skin, tendons, and cartilage^5^. Vimentin is an intermediate filament protein involved in maintaining cellular structure and supporting tissue integrity^8^. Citrullinated vimentin has been predominantly studied as an autoantigen in RA and is elevated compared to controls^9^. However, there is emerging evidence of its potential role in PsA and PsO, suggesting its involvement in the inflammatory and tissue remodeling processes associated with these diseases^10^.

An altered ECM turnover leads to tissue inflammation, destruction, and fibrosis. The persistent strain leads to higher recruitment of immune cells which increase the concentration of degradative enzymes, such as metalloproteinases (MMP) and aggrecanases, resulting in a vicious cycle of tissue remodeling of the surrounding ECM in the various tissues^11^. Several MMPs play a role in tissue degradation: MMP-1 degrades type I, II, III collagen; MMP-3 degrades type III collagen; MMP-9 degrades type IV and V and VI collagens and MMP-13 degrades type I, II, and III collagen^11,12^. Degradation and formation metabolites of these ECM proteins have been studied in RA, PsA and PsO previously, and found to be elevated in both joint and skin disorders^3,4,13,14^. More specifically, higher levels of MMP-1, -3, -9 and -13 have been observed in RA^15^ while only MMP-1 and -3 showed increased levels in psoriatic patients^16,17^. In RA, type I (C1M), type II (C2M), type III (C3M), and type VI (C6M) collagen degradation biomarkers have shown increased levels, indicating elevated collagen degradation and turnover^18–20^. In PsA, C1M, C2M, C3M, type IV (C4M) collagen degradation, C6M and type III (PRO-C3) and type VI (PRO-C6) collagen formation biomarkers were elevated compared to controls^3,14,21^. Lastly, in PsO, PRO-C3 and C2M were elevated compared to controls, while PRO-C1 and C3M were lowered^3^.

The quantitative assessment of changes in blood biomarker levels that reflect alterations at the tissue level has the potential to serve as a biomarker to assist in the diagnosis (especially in the early phases of disease manifestation) and monitoring of disease. Therefore, it is essential to identify biomarkers that are pathology-relevant and reflect treatment effect or disease flares.

Besides ECM biomarkers, biomarkers of patient’s functional state are of great importance for disease monitoring. Inflammatory arthritis often affects the small joints in the hands, leading to hand impairments as a major cause of disability in these patients^22^ that can be quantified by objective functional tests^23^. Grip strength, extensively studied in RA, is a valuable tool for predicting disease progression^24^. Fine motor skills are important for maintaining adequate hand function and can be objectively measured with the Moberg Picking-Up Test (MPUT)^25,26^. Previous studies indicated a similar decline in hand function in PsA and PsO^23^. In RA patients, functional impairments could already be related to clinical scores like the Disease Activity Score 28 (DAS-28)^27^. Evaluating the functional state of patients enables the grading of disease burden and the impact of the disease on quality of life, and may be indicative of subclinical alterations^23^. Understanding and measuring functional status is crucial for personalized treatment approaches. While it has been demonstrated that functional scores can be related with subclinical inflammation (by MRI or ultrasonography^28^), it is not well-studied how changes in ECM tissue turnover relates with functional status in inflammatory arthritis.

In this observational cross-sectional study, we aimed to investigate differences in biomarker concentrations of ECM formation and degradation, together with measures of hand function impairment between patients with RA, PsA and PsO and controls. Furthermore, we explored the relationship between the ECM biomarkers, hand function and clinical scores.

## Materials and methods

### Study participants

This analysis includes data from patients and non-arthritic control subjects (controls) of three cross-sectional studies conducted in the outpatient clinics of the Department of Internal Medicine 3, Universitätsklinikum Erlangen, Germany. For study 1 and 2 (ethical approval #125_16B), patients diagnosed with RA (American College of Rheumatology/European League Against Rheumatism (ACR/EULAR) 2010 criteria^29^, study 1 only), PsA (Classification Criteria for Psoriatic Arthritis (CASPAR)^30^) and PsO (without any signs of arthritis, dactylitis, enthesitis or inflammatory back pain) were included. Controls were locally recruited from an existing cohort^31^ and using social platforms, flyers and personal conversation (study 3 ethical approval #357_20B). Exclusion criteria for all participants were injuries or fractures of the hand joints within the last five years, disorders of the musculoskeletal system beyond the rheumatic disease, gross bony deformities of the hands and sign of psoriasis (controls only). All assessments included in this analysis were undertaken at the same day and performed identically in all three studies.

### Clinical data and patient-reported outcomes

Demographic data, including age, sex, and body mass index (BMI) were recorded for all participants. All participants underwent a standardized 68 tender (TJC) and 66 swollen joint count (SJC) by a trained physician. C-reactive protein (CRP) was analyzed from serum samples by standard clinical procedures. Data on disease, disease activity and treatment were retrospectively retrieved from patient records for the day of assessment. Current treatment and treatment history were categorized with respect to history of biological treatments, i.e. biologic naïve or as undergoing treatment with biological or targeted Disease-Modifying-Anti-Inflammatory Drugs (DMARDs) and/or taking Glucocorticoids. All participants reported perceived physical function (health assessment questionnaire (HAQ)), pain (visual analogue scale (VAS) for pain), quality of life (Short Form 36 Health Survey Questionnaire (SF-36))^32^ and physical activity (International Physical Activity Questionnaire (IPAQ)^33^).

### ECM biomarkers

Blood samples were drawn on the day of clinical data collection. Samples were allowed to clot at room temperature for at least 30 min, centrifuged and serum was aliquoted and frozen at -80°C until analysis. ECM turnover biomarkers of type I (C1M), II (C2M), III (C3M), IV (C4M) and VI (PRO-C6) collagen degradation, type IV collagen 7S domain (PRO-C4), type I (PRO-C1), III (PRO-C3) and VI (PRO-C6) collagen formation, aggrecan degradation (ARG), degraded prolargin (PROM) and citrullinated and degraded vimentin (VICM) were measured using validated manual or automated enzyme-linked immunosorbent assays (ELISAs). Assays were either colorimetry or chemiluminescence-based and information on assay development, validation parameters, and references to their technical papers are summarized in Table 1. Sample measurements were conducted in duplicates, and samples with a coefficient of variation (CV) > 15% were re-run.

**Table 1:** Extracellular biomarkers measured in the study.

Biomarker	Description of the biomarker	Implication	Reference
C1M	MMP-2/9/13-degraded type I collagen	Interstitial matrix degradation	^34^
C2M	MMP (multiple) -degraded type II collagen	Cartilage degradation	^35^
ARG	ADAMTS-4/5-cleaved aggrecan	Cartilage degradation	^36^
C3M	MMP-9-degraded type III collagen	Interstitial matrix degradation	^37^
C4M	MMP (multiple)-degraded type IV collagen	Primarily basal lamina disruption	^38^
C6M	MMP-2/9-degraded type VI collagen	Microfibril degradation	^39^
VICM	Citrullinated and MMP-degraded vimentin	Inflammation	^40^
PRO-C1	Type I collagen N-terminal propeptide	Bone formation	^41^
PRO-C3	Type II collagen N-terminal propeptide	Fibrosis	^42^
PRO-C4	Type IV 7S domain collagen	Basement membrane turnover	^43^
PRO-C6	Type VI collagen, alpha-3 chain, C5 domain	Fibrosis	^44^

### Hand function tests

Fine motor skills were assessed by the MPUT according to a standardized protocol^25^. Participants picked up and transported 12 small objects into a little box while task completion (seconds) was recorded. The test was performed twice with each hand and the fastest trials for each hand were used for the analysis. Isometric grip strength (lbs) was measured with a hand dynamometer (Lafayette Instrument, Lafayette, IN, USA). For each hand the highest attempt out of three trials was used for the analysis. Perceived hand function was recorded by the Michigan Hand Questoinnaire (MHQ): based on 6 domains (overall hand function, activities of daily living (ADLs), pain, work performance, aesthetics, and satisfaction with hand function) a total MHQ score was calculated (range: 0 (poorest function) to 100 (ideal function))^45^.

### Statistical analysis

Baseline characteristics are described as number (frequency) and percentage for categorical variables, and as mean ± standard deviation (SD) for continuous variables. A heatmap analysis was conducted to visualize the hierarchical clustering of the biomarkers and to categorize groups of biomarkers based on their relative proximity in the heatmap. Kruskal–Wallis rank test was used to examine baseline differences between groups and between each diseased group and controls. The biomarker data was natural log-transformed for normalization. Linear regression models using robust standard errors were performed to compare biomarker levels between groups while controlling for the effects of the demographic confounders age, sex and BMI. Targeting an overall type-1 error rate of 5%, the significance threshold for each comparison was set at 0.004 considering that in total 12 biomarkers were analyzed. The p-values from post-hoc comparisons between groups were adjusted using Holm’s method.

Spearman’s rank correlation coefficients were calculated to assess the relationships between biomarker levels, hand function and clinical scores. The p-values were adjusted using the false discovery rate method, with significance considered below an adjusted value of 0.05. For all regression models and correlations full-case analysis was performed.

Statistical analysis was performed with R (version 4.2.2, R Foundation for Statistical Computing, Vienna, Austria) and figures were designed using GraphPad Prism version 9.5.1 (GraphPad Software, San Diego, California, USA).

## Results

### Study participant characteristics

A total of 412 subjects were included in this study (RA, n = 85; PsA, n = 115; PsO, n = 102; controls, n = 110). Participant characteristics are summarized in Table 2.

**Table 2:** Summary of subject characteristics and clinical data.

	RA (N = 85)	PsA (N = 115)	PsO (N = 102)	Control (N = 110)	p value
Age	58.4 (13.2)‡	53.7 (12.1)‡	45.8 (14.4)	46.6 (18.1)	< 0.001
Sex, male, n(%)	30 (35.3%)	57 (49.6%)	62 (60.8%)†	49 (45.0%)	0.006
BMI, kg/m2	27.3 (5.2)‡	29.4 (6.6)‡	28.8 (6.5)‡	24.7 (4.8)	< 0.001
CRP, mg/l	7.2 (12.3)‡	7.1 (12.8)‡	6.0 (5.6)‡	3.2 (3.0)	< 0.001
Disease duration, years	10.8 (10.1)	9.1 (9.7)	12.2 (11.4)	NA	NA
VAS pain, mm	33.4 (24.2)‡	35.8 (27.2)‡	25.5 (26.0)‡	4.3 (12.2)	< 0.001
HAQ score (0–3 units)	0.9 (0.6)‡	0.6 (0.6)‡	0.4 (0.6)‡	0.0 (0.2)	< 0.001
IPAQ total walking, MET-min/week	1700.1 (2203.1)	2217.5 (3021.4)	989.5 (1605.3) ‡	988.3 (1272.0)	0.001
IPAQ total medium, MET-min/week	3504.5 (4365.7)	4018.9 (4499.6)	2926.6 (4260.3)	2793.4 (3360.0)	0.123
IPAQ total sitting, min/week	2094.4 (1150.3)	2189.6 (1298.2)	2349.1 (1321.9)	2437.6 (1401.4)	0.153
MHQ dominant (0–100 units)	63.9 (14.3)‡	70.1 (15.6)‡	76.1 (16.6)‡	88.6 (10.3)	< 0.001
MHQ non dominant (0–100 units)	63.3 (9.7)‡	66.7 (9.2)‡	68.5 (10.4)‡	85.9 (12.2)	< 0.001
RAID score (0–10 units)	2.9 (1.7)	NA	NA	NA	NA
DLQI score (0–30 units)	NA	3.1 (3.9)‡	8.8 (7.1)‡	0.7 (1.5)	NA
PSAID score (0–20 units)	NA	5.2 (4.0)	4.5 (5.3)	NA	NA
Grip strength dominant hand, pounds	56.5 (32.6)‡	72.8 (35.0)‡	88.4 (36.4)	89.3 (25.3)	< 0.001
Grip strength non dominant hand, pounds	59.0 (31.0)‡	72.7 (32.4)‡	80.3 (26.2)‡	91.2 (18.8)	< 0.001
MPUT dominant hand, seconds	16.9 (7.3)‡	16.0 (7.7)‡	14.7 (5.0)‡	10.4 (2.1)	< 0.001
MPUT non dominant hand, seconds	20.3 (41.7)‡	17.0 (13.2)‡	14.5 (4.7)‡	10.8 (2.0)	< 0.001
MASES (0–13 units)	NA	1.4 (2.3)‡	1.2 (2.7)‡	0.1 (0.3)	NA
PASI score (0–72 units)	NA	1.4 (2.7)	3.3 (4.2)	NA	NA
Tender joint count 78, n	5.4 (7.0)‡	5.4 (7.7)‡	1.8 (4.8)‡	0.2 (1.0)	< 0.001
Swollen joint count, 76, n	0.6 (1.1) ‡	0.7 (1.9) ‡	0.2 (1.0) †	0.0 (0.1)	< 0.001
Tender joint count 28, n	3.6 (4.3)‡	3.9 (5.4)‡	1.7 (3.4)‡	0.2 (0.7)	< 0.001
Swollen joint count 28, n	0.7 (1.4)‡	0.5 (1.3)‡	0.0 (0.2)	0.0 (0.1)	< 0.001
DAS-28 total (0–10 units)	3.0 (1.3)‡	2.9 (1.4)‡	2.2 (1.2)‡	1.6 (0.7)	< 0.001
ksk36 (0–100 units)	38.0 (9.7)‡	41.6 (11.3)‡	46.1 (10.5)‡	54.0 (6.1)	< 0.001
psk36 (0–100 units)	46.2 (11.9)	48.3 (11.0)‡	45.6 (11.4)‡	52.2 (8.2)	< 0.001
Treatment					
Biological naïve, n (%)	11 (13.1%)	29 (27.6%)	52 (57.1%)	NA	
Biological DMARDs, n (%)	66 (80.5%)	64 (62.1%)	31 (34.4%)	NA	
Targeted DMARDs, n (%)	2 (2.6%)	3 (3.0%)	2 (2.2%)	NA	
Glucocorticoids, n (%)	34 (41.5%)	10 (10.0%)	4 (4.4%)	NA	

*BMI* body mass index, *CRP* C-reactive protein, *VAS* visual analogue scale, *HAQ* Health Assessment Questionnaire, *IPAQ* International Physical Activity Questionnaire, *MHQ* Michigan Hand Questionnaire, *RAID* Rheumatoid Arthritis Impact of Disease Score, *DLQI* Dermatology Life Quality Index, *PSAID* Psoriatic Arthritis Impact of Disease, *MPUT* Moberg Picking-Up Test, *MASES* Maastricht Ankylosing Spondylitis Enthesitis Score, *PASI* Psoriasis Area Severity Index, *DAS* Disease Activity Score, *ksk36* physical scale of Short Form 36 Health Survey Questionnaire (SF-36), *psk36* psychological scale of SF-36, *DMARDs* disease modified anti-rheumatic drugs.

Except where indicated otherwise, mean ± SD is presented. Kruskal–Wallis rank test was used. Each diseased group was further compared with the control group. Significant differences are indicated as † 0.01 ≤ *p* < *0.05*, ‡*p* < *0.01*. Biomarker and hand function scores missing data can be found in Supplementary Table S1.

At the time of assessment, the majority of the RA and PsA patients were under standard of care treatment with biological or targeted DMARDS and/or Glucocorticoids; more than 80% of the RA and 60% of the PsA patients were treated with biological DMARDs. More RA patients were on Glucocorticoid treatment compared with PsA and PsO patients. Approximately 60% of PsO patients had no history of biological treatments. Most RA and PsA patients were in clinical remission or with mild disease activity (DAS-28 3.0 (1.3), 2.9 (1.4); PASI 1.4 (2.7) respectively) at the time of data collection.

For both, the dominant and non-dominant hand, all patients showed slower MPUT performance, lower grip strength and more perceived hand impairment by MHQ compared to controls. Within the patient groups, RA patients showed the worst hand impairment followed by PsA and PsO. In RA patients grip strength was lower in the dominant hand compared with the non-dominant hand; for all other groups the dominant hand was the stronger one. The MPUT times and MHQ scores did not differ between non-dominant and dominant hand within the groups (Supplementary Table S2).

### ECM turnover biomarkers in RA, PsA and PsO

**Fig. 1:**
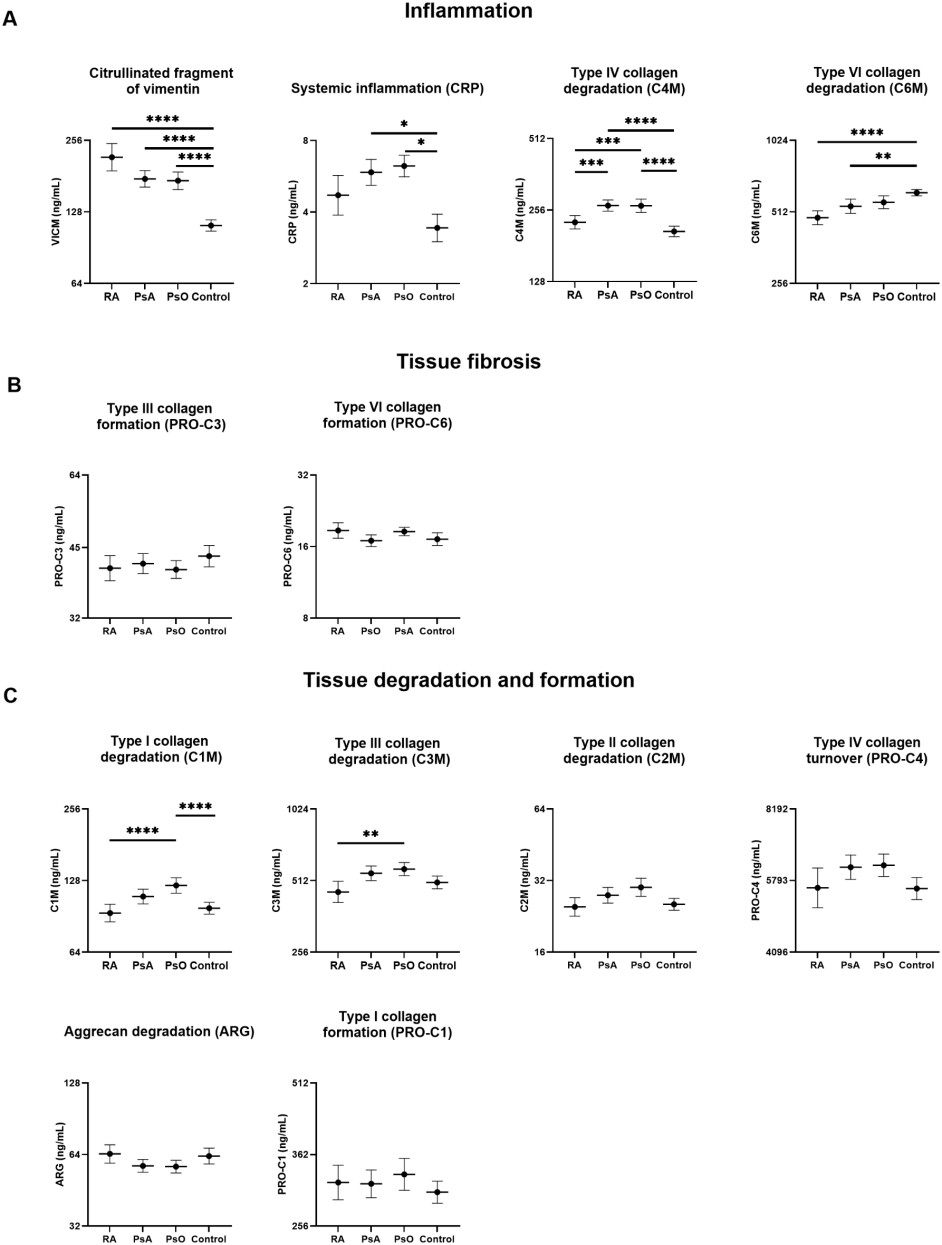
Extracellular matrix remodeling biomarker levels in serum samples from patients with RA (N = 85), PsA (N = 115), PsO (N = 102) and controls (N = 110). Linear regression models using robust standard errors were performed to compare biomarker levels within the groups with age, sex and BMI as covariates. Y axis is in log2 scale and values are shown as the estimated mean with 95% confidence intervals. P-values were adjusted by Holm correction and significance threshold was set at 0.004 considering that 12 biomarkers were analyzed. Significance is shown as ** p < 0.004, ***p < 0.001 and ****p < 0.0001. Raw biomarker values for each group are provided in Supplementary Figure S1.

Serum concentrations of ECM biomarkers and group difference for RA, PsA, PsO and controls are summarized in Fig. 1. Absolute biomarker levels for each group and comparison of linear regression models using robust standard errors with adjustments for age, sex and BMI to those without adjustments are presented in Supplementary Table S3.

The heat map analysis shows the hierarchical clustering of the biomarkers (Supplementary Figure S2). Subsequently, the biomarkers were categorized into three groups based on their relative proximity in the heatmap: inflammation (VICM, C4M, C6M and CRP), tissue degradation and formation (C3M, PRO-C4, C2M, C1M, ARG and PRO-C1), and tissue fibrosis (PRO-C3 and PRO-C6).

Levels of the inflammation marker VICM were higher in RA, PsA, and PsO compared to controls (Fig. 1A, p < 0.0001). PsA and PsO showed significantly higher C4M levels compared to controls (p < 0.0001, p < 0.0001), while C6M was lower in patients with RA, PsA and PsO than in controls (p < 0.0001, p < 0.001 and p < 0.01, Fig. 1A). The tissue fibrosis markers, PRO-C3 and PRO-C6, did not present any significant differences among the groups (Fig. 1B). The tissue turnover biomarkers C1M presented higher levels in PsO compared to controls and RA (Fig. 1C, p < 0.004, p < 0.0001, respectively). C2M and PRO-C4 showed higher levels in PsA and PsO compared to RA and controls but did not reach statistical significance (Fig. 1C). ARG and PRO-C1 presented similar levels and no significant differences across the different groups (Fig. 1C).

### Association between ECM biomarkers, clinical features, and hand function in patients with RA, PsA and PsO

**Fig. 2:**
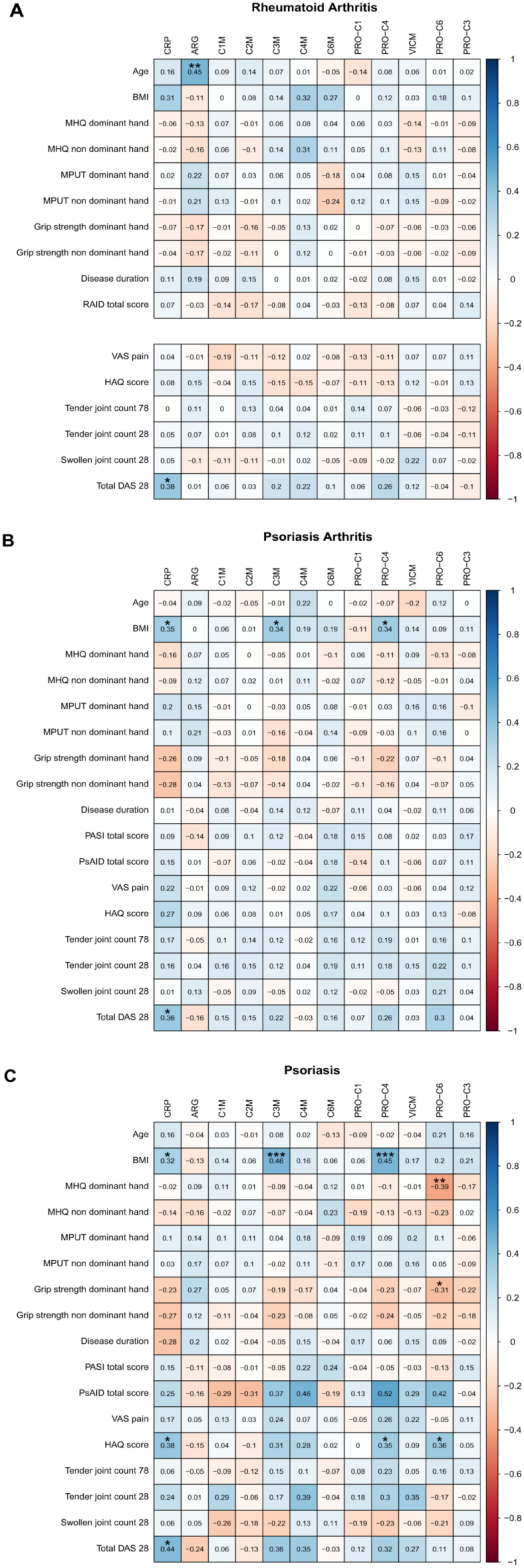
Spearman’s correlation between serological biomarkers and clinical scores were performed. Spearman’s rho (ρ) is shown. Significance of correlations are shown as * p < 0.05, **p < 0.01, and ***p < 0.001. *BMI* body mass index, *VAS* pain visual analogue scale pain, *HAQ* Health Assessment Questionnaire, *IPAQ* International Physical Activity Questionnaire, *RAID* Rheumatoid Arthritis Impact of Disease Score, *PSAID* Psoriatic Arthritis Impact of Disease, *MPUT* Moberg Picking-Up Test, *PASI* Psoriasis Area Severity Index, *DAS* Disease Activity Score, *ksk36* physical scale of Short Form 36 Health Survey Questionnaire (SF-36), *psk36* psychological scale of SF-36, *MHQ* Michigan Hand Questionnaire, *CRP* C-reactive protein, *ARG* aggrecan *ADAMTS* degradation, *C1M* MMP-2/9/13-degraded type I collagen, *C2M* MMP (multiple) -degraded type II collagen, *C3M* MMP-9-degraded type III collagen, *C4M* MMP (multiple)-degraded type IV collagen, *C6M* MMP (multiple)-degraded type IV collagen, *PRO-C1* Type I collagen N-terminal propeptide, *PRO-C4* Type IV 7S domain collagen, *VICM* citrullinated and MMP-degraded vimentin, *PRO-C6* Type VI collagen, alpha-3 chain, C5 domain, *PRO-C3* type II collagen N-terminal propeptide.

In patients with RA and PsA, we observed weak and non-significant correlations between the ECM biomarkers and the hand function scores (all *ρ* < 0.3, Fig. 2A and 2B). However, among patients with PsO, we found that PRO-C6 displayed a negative correlation with the MHQ Score and grip strength of the dominant hand (*ρ* = -0.39 and -0.31; p < 0.01 and p < 0.05, respectively, Fig. 2C).

We did not find any significant correlations between the specific disease activity scores (DAS-28, Rheumatoid Arthritis Impact of Disease Score (RAID), Psoriatic Arthritis Impact of Disease (PSAID), Psoriasis Area Severity Index (PASI)) and the ECM biomarkers in any of the groups. PRO-C4 and PRO-C6 moderately correlated with HAQ in PsO patients (*ρ* = -0.35 and 0.36; all p < 0.05, respectively, Fig. 2C).

## Discussion

The present study evaluated differences in ECM turnover biomarker levels between patients with RA, PsA, PsO and controls, and the relationship of the biomarker levels with hand function impairments measured by objective and subjective scores. Overall, our results suggested that patients with PsA and PsO had higher tissue degradation and inflammation compared to patients with RA (under well-controlled disease) and controls, whereas RA patients presented with the worst hand function impairments among study groups. Furthermore, ECM biomarkers correlated more frequently and stronger with hand function scores in patients with PsO compared with RA and PsA, while no significant correlations were found for clinical disease activity scores and ECM biomarkers in any of the diseased groups.

In this study, concentrations of MMP-degraded fragments of citrullinated vimentin, type I and IV collagen, reflected by VICM, C1M and C4M, respectively, were higher in patients with RA, PsA and PsO, compared with controls. In agreement with our findings, previous studies have shown increased levels of C1M, C4M and VICM in RA compared to controls^4,9^, elevated levels of C1M and C4M in PsA^3,14,46,47^ and in PsO^3,47^. We found that C6M (MMP-degraded type VI collagen) was higher in controls than in RA, PsA and PsO.

When comparing the biomarker levels within the diseased groups, we observed that patients with PsA and PsO had higher levels of the catabolic markers (C1M and C4M) compared with RA, but only significant for C1M. In our cohort, the RA group had the highest number of patients treated with bDMARDs (N = 66, 80.5%) and at the same time presented with the lowest C1M levels. This is consistent with previous studies that showed that biomarkers reflecting type I, II, III, IV and VI collagen degradation, C1M, C2M, C3M, C4M and C6M, respectively, were modulated by treatment (Janus kinase inhibitor^4^, IL-6 inhibitors^18,48^, or methotrexate^49^) in patients with RA, resulting in lower biomarker concentrations independently of disease activity. Another study showed that serum levels of C1M, C3M C4M and C6M were also decreased in PsA patients treated with interleukin-23p19-subunit monoclonal antibody, and those biomarkers were higher compared to controls at baseline^14^.

No differences in the catabolic markers were found between the psoriatic diseases. In contrast to our findings, Holm Nielsen et al.^3^ found that C1M was able to differentiate between PsA and PsO, with PsA patients having higher levels of the biomarker. However, the mentioned study had a smaller sample size and patients with PsA had higher skin disease activity (reflected by PASI score of 2.8). On the other hand, they also found no differences in C2M, C3M and C4M between the two diseases. Other ECM related proteins have been studied previously, and a combination of CRP, MMP-3 and type II collagen (CPII, C2C) biomarkers were able to distinguish patients with PsA from those with PsO^47^. The study of Groen et al.^47^ did not report current treatment of the included PsA patients, which might be causing the differences in the ECM biomarker levels between the studies. In addition, while the PsO patients included in this study had no inflammatory arthritis at the time of data collection, they had been referred to a rheumatologist to exclude psoriasis arthritis. Therefore, the role of catabolic biomarkers as early predictors for the transition from PsO to PsA should be further studied in prospective trials specifically designed for the purpose to further elucidate their potential to indicate disease onset.

In this study we did not find an association between anabolic or fibrotic markers (reflected by PRO-C1, PRO-C3 and PRO-C6) and the studied diseases. However, lower PRO-C1 levels have been previously reported in PsA compared to healthy subjects^3^. Regarding the levels of PRO-C3 and PRO-C6 in these diseases, previous study results are inconclusive: Schett et al.^14^ observed no differences in PRO-C3 and PRO-C6 levels between PsA patients and controls, whereas Holm Nielsen et al. observed higher levels of PRO-C3 in PsO compared to controls, where the patients with PsO had high disease activity (PASI score of 8.1)^3^. These results indicate that catabolic activity is clearly associated with RA, PsA and PsO whereas fibrotic or anabolic activity needs to be further elucidated in these diseases.

To our knowledge, this is the first study investigating the association between a panel of ECM biomarkers and hand function impairment to better understand the relationship between tissue-related changes and functional impairments of the musculoskeletal system. While it is well known that hand function is highly impaired in RA, Liphardt et al.^23^ found that hand function was also altered in patients with PsO. In this study, PRO-C6 was negatively correlated to grip strength and perceived hand function (MHQ) in PsO patients, meaning that high type VI collagen formation might contribute to a worsening in hand function and weaker grip strength, hence greater functional impairment. Previous studies have already established a link between PRO-C6 and increased arterial stiffness in patients with type 1 diabetes^44^. Considering that PsO patients are at high risk of developing arterial stiffness^50^, which could potentially be reflected in a decrease in grip strength, this functional parameter is an important factor in the progression of PsO. In this study, we observed only weak correlations between the ECM biomarkers and hand function scores in patients with RA and PsA. As discussed above, treatment likely modulates biomarker levels of the well-treated patients and in low disease activity individual variation in hand functions may be greater than in active disease, both resulting in weak associations between disease activity and the state of functional impairment with ECM biomarkers in this cross-sectional analysis. Our findings suggest that hand impairment is present in both, acute inflammation and as a result of chronic tissue deterioration. The association of hand function with tissue turnover by ECM biomarkers may be prominent in acute, untreated disease activity.

In this study we did not observe significant correlations between clinical scores (RAID or DAS-28) and the ECM biomarker concentrations. This may be due to the target-driven treatment strategy resulting in a narrow range of disease activity in this cohort, which may impede our ability to observe such an association even if it exists. Indeed, other studies have shown that, C1M and C3M correlated with DAS28, joint counts and HAQ^6^ in patients with RA and when treated with methotrexate, changes in biomarker levels of C1M, C3M, C4M and CRPM correlate with change in disease activity after 8 weeks of treatment also in RA patients^18^. Further studies should therefore focus on long-term follow up to investigate intra-individual disease activity and treatment-related changes for both ECM biomarkers and hand function in the different patient groups of RA, PsA and PsO. Additionally, this would allow to quantify how the patient specific change in hand function is related to change in ECM biomarkers and provide insights on the extent to which they are linked.

Some limitations were encountered in this study. Firstly, there was a large inter-individual variation of biomarker levels which we tried to minimize by using linear regression models and robust standard errors. Secondly, out of the three patient groups, all RA and most of the PsA patients were under well-regulated standard of care treatment with the already discussed effects on ECM biomarker concentrations. The cross-sectional data set included current treatment and treatment history which we reported in the subject characteristics, but the study design did not allow for a detailed sub-analysis of a treatment effect on the investigated associations. These limitations highlight the need for longitudinal studies in large cohorts and in the context of treatment to validate our findings and gain deeper understanding of the ECM remodeling and hand function impairment. In addition, the impact of adjusting for confounders on biomarker levels, in particular BMI, as one disease characteristic in Pso and PsA patients, needs to be further investigated.

## Conclusion

In conclusion, this study showed that patients with RA, PsA, and PsO had significant alterations in the ECM as quantified by biomarkers of citrullinate vimentin degradation, type I, III, IV and VI collagen degradation. However, it is important to consider that blood-based biomarkers are highly dynamic and dependent on treatment, and RA and PsA patients were in a relatively low disease activity state due to current targeted-treatment strategies. Weak associations were found between the blood biomarker levels and quantitative hand function, especially in the PsO group including the highest number of patients with no history of biological treatment. This underlines that both markers reflect different pathological processes and a possible causal relationship between the change in these markers need to be further explored with longitudinal data. While blood-based biomarkers reflect tissue turnover, hand function impairments mirror the overall disability caused by the disease progression, which is present even in patients with low to moderate disease activity. The use of blood-based biomarkers can help to identify patients at risk to develop inflammatory arthritis and hand function is well suited to grade functional impairment in addition to common clinical scores.

## Supplementary Information

Supplementary Information.

## Data Availability

The datasets used and/or analysed during the current study are available from the corresponding author on reasonable request.

## References

[1] McInnes, I. B. & Schett, G. The pathogenesis of rheumatoid arthritis. *N. Engl. J. Med.* **55**, 2255–2270 (2011). 10.1056/NEJMra1004965 22150039

[2] Ritchlin, C. T., Colbert, R. A. & Gladman, D. D. Psoriatic Arthritis. *N. Engl. J. Med.* **376**, 957–70 (2017). 28273019 10.1056/NEJMra1505557

[3] Holm Nielsen, S. et al. Differentiating patients with psoriasis from psoriatic arthritis using collagen biomarkers. *Clin. Exp. Rheumatol.*10.55563/clinexprheumatol/jmt9jv (2022). 35916294 10.55563/clinexprheumatol/jmt9jv

[4] Thudium, C. S. et al. The Janus kinase 1/2 inhibitor baricitinib reduces biomarkers of joint destruction in moderate to severe rheumatoid arthritis. *Arthritis. Res. Ther.* **22**, 1–11 (2020). 33046136 10.1186/s13075-020-02340-7 PMC7552555

[5] Karsdal, M. A. Type I collagen. In *Biochemistry of Collagens, Laminins and Elastin: Structure, Function and Biomarkers* (ed. Karsdal, M. A.) 1–389 (Elsevier, 2019).

[6] Maijer, K. I. et al. Neo-epitopes-fragments of cartilage and connective tissue degradation in early rheumatoid arthritis and unclassified arthritis. *PLoS ONE* **11**, 1–12 (2016). 10.1371/journal.pone.0149329 PMC4809616 27019199

[7] Yurchenco, P. D., Amenta, P. S. & Patton, B. L. Basement membrane assembly, stability and activities observed through a developmental lens. *Matrix Biol.* **22**, 521–538 (2004). 14996432 10.1016/j.matbio.2003.10.006

[8] Danielsson, F. et al. Vimentin diversity in health and disease. *Cells* **7**, 1–38 (2018). 10.3390/cells7100147 PMC6210396 30248895

[9] Drobinski, P. J. et al. In contrast to Anti-CCP, MMP-degraded and citrullinated vimentin (VICM) is both a diagnostic and a treatment response biomarker. *Int. J. Mol. Sci.* **24**, 1–12 (2022). 36613765 10.3390/ijms24010321 PMC9820189

[10] Groen SS, Nielsen SH, Bay-Jensen AC, et al. Are Serological Protease-mediated Peptides of Tissue Remodeling and Inflammation the New Disease Activity Biomarkers in Psoriatic Arthritis Patients? [abstract]. Arthritis Rheumatol. 74 (2022).

[11] Siebuhr, A. S. et al. Matrix Metalloproteinase-dependent turnover of cartilage, synovial membrane, and connective tissue is elevated in rats with collagen induced arthritis. *J. Transl. Med.*10.1186/1479-5876-10-195 (2012). 22992383 10.1186/1479-5876-10-195 PMC3551788

[12] Grillet, B. et al. Matrix metalloproteinases in arthritis: Towards precision medicine. *Nat. Rev. Rheumatol.* **19**, 363–377 (2023). 37161083 10.1038/s41584-023-00966-w

[13] Bay-Jensen, A. C. et al. Tissue metabolite of type I collagen, C1M, and CRP predicts structural progression of rheumatoid arthritis. *BMC Rheumatol.* **3**, 1–10 (2019). 10.1186/s41927-019-0052-0 PMC6390574 30886991

[14] Schett, G. et al. Collagen turnover biomarkers associate with active psoriatic arthritis and decrease with guselkumab treatment in a phase 3 clinical trial (DISCOVER-2). *Rheumatol. Ther.* **9**, 1017–1030 (2022). 35352313 10.1007/s40744-022-00444-x PMC9314487

[15] Kim, K. S. et al. Expression levels and association of gelatinases MMP-2 and MMP-9 and collagenases MMP-1 and MMP-13 with VEGF in synovial fluid of patients with arthritis. *Rheumatol. Int.* **31**, 543–547 (2011). 20665024 10.1007/s00296-010-1592-1

[16] Jadon, D. R. et al. Serum bone-turnover biomarkers are associated with the occurrence of peripheral and axial arthritis in psoriatic disease: A prospective cross-sectional comparative study. *Arthritis Res. Ther.* **19**, 1–10 (2017). 28934972 10.1186/s13075-017-1417-7 PMC5609020

[17] Mezentsev, A., Nikolaev, A. & Bruskin, S. Matrix metalloproteinases and their role in psoriasis. *Gene* **540**, 1–10 (2014). 24518811 10.1016/j.gene.2014.01.068

[18] Drobinski, P. J. et al. Connective tissue remodelling is differently modulated by tocilizumab versus methotrexate monotherapy in patients with early rheumatoid arthritis: the AMBITION study. *Arthritis Res. Ther.* **23**, 1–12 (2021). 33413588 10.1186/s13075-020-02378-7 PMC7789531

[19] Thudium, C. S. et al. Bone phenotypes in rheumatology—There is more to bone than just bone. *BMC Musculoskelet. Disord.* **21**, 1–20 (2020). 10.1186/s12891-020-03804-2 PMC7700716 33248451

[20] Kjelgaard-Petersen, C. F. et al. Translational biomarkers and ex vivo models of joint tissues as a tool for drug development in rheumatoid arthritis. *Arthritis Rheumatol.* **70**, 1419–1428 (2018). 29669391 10.1002/art.40527 PMC6174937

[21] Bay-Jensen, A. C. et al. POS0006 identification of fibrotic and fibrolytic endotypes in rheumatic disease cohorts. *Ann. Rheum. Dis.* **81**, 216 (2022).

[22] Bodur, H., Yilmaz, O. & Keskin, D. Hand disability and related variables in patients with rheumatoid arthritis. *Rheumatol. Int.* **26**, 541–4 (2006). 16079993 10.1007/s00296-005-0023-1

[23] Liphardt, A.-M. et al. Similar impact of psoriatic arthritis and rheumatoid arthritis on objective and subjective parameters of hand function. *ACR Open Rheumatol.* **2**, 734–40 (2020). 33241646 10.1002/acr2.11196 PMC7738802

[24] Rydholm, M. et al. The relation between disease activity, patient-reported outcomes, and grip force over time in early rheumatoid arthritis. *ACR Open Rheumatol.* **1**, 507–15 (2019). 31777832 10.1002/acr2.11062 PMC6857997

[25] Stamm, T. A. et al. Moberg picking-up test in patients with inflammatory joint diseases: a survey of suitability in comparison with button test and measures of disease activity. *Arthritis Rheum.* **49**, 626–32 (2003). 14558047 10.1002/art.11378

[26] Ng, C. L., Ho, D. D. & Chow, S. P. The Moberg pickup test: Results of testing with a standard protocol. *J. Hand Ther.* **12**, 309–312 (1999). 10622197 10.1016/s0894-1130(99)80069-6

[27] Durmus, D. et al. Michigan Hand Outcomes Questionnaire in rheumatoid arthritis patients: Relationship with disease activity, quality of life, and handgrip strength. *J. Back Musculoskelet. Rehabil.* **26**, 467–73 (2013). 23948837 10.3233/BMR-130408

[28] Ozer, P. K. et al. Ultrasound-defined remission for good functional status in rheumatoid arthritis. *Indian J. Med. Res.* **146**, 230–6 (2017). 29265024 10.4103/ijmr.IJMR_548_15 PMC5761033

[29] Aletaha, D. et al. 2010 Rheumatoid arthritis classification criteria: an American College of Rheumatology/European League Against Rheumatism collaborative initiative. *Arthritis Rheum.* **62**, 2569–81 (2010). 20872595 10.1002/art.27584

[30] Taylor, W. et al. Classification criteria for psoriatic arthritis: development of new criteria from a large international study. *Arthritis Rheum.* **54**, 2665–73 (2006). 16871531 10.1002/art.21972

[31] Berlin, A. et al. The ageing joint-standard age- and sex-related values of bone erosions and osteophytes in the hand joints of healthy individuals. *Osteoarthr. Cartil.* **27**, 1043–7 (2019). 10.1016/j.joca.2019.01.019 30890457

[32] Ware, J. E. et al. *Conceptualization and Measurement of Health for Adults in the Health Insurance Study: Vol. I, Model of Health and Methodology* (Rand Corporation, 1980).

[33] Craig, C. L. et al. International physical activity questionnaire: 12-country reliability and validity. *Med. Sci. Sport Exerc.* **35**, 1381–95 (2003). 10.1249/01.MSS.0000078924.61453.FB 12900694

[34] Leeming, D. J. et al. A novel marker for assessment of liver matrix remodeling: An enzyme-linked immunosorbent assay (ELISA) detecting a MMP generated type I collagen neo-epitope (C1M). *Biomarkers* **16**, 616–628 (2011). 21988680 10.3109/1354750X.2011.620628

[35] Bay-Jensen, A.-C. et al. Enzyme-linked immunosorbent assay (ELISAs) for metalloproteinase derived type II collagen neoepitope, CIIM–increased serum CIIM in subjects with severe radiographic osteoarthritis. *Clin. Biochem.* **44**, 423–429 (2011). 21223960 10.1016/j.clinbiochem.2011.01.001

[36] He, Y. et al. Development of a highly sensitive chemiluminescence immunoassay for quantification of aggrecanase-generated ARGS aggrecan fragments in serum. *Osteoarthr. Cartil. Open* **3**, 100162 (2021). 36474987 10.1016/j.ocarto.2021.100162 PMC9718149

[37] Barascuk, N. et al. A novel assay for extracellular matrix remodeling associated with liver fibrosis: An enzyme-linked immunosorbent assay (ELISA) for a MMP-9 proteolytically revealed neo-epitope of type III collagen. *Clin. Biochem.* **43**, 899–904 (2010). 20380828 10.1016/j.clinbiochem.2010.03.012

[38] Veidal, S. S. et al. Assessment of proteolytic degradation of the basement membrane: A fragment of type IV collagen as a biochemical marker for liver fibrosis. *Fibrogenesis Tissue Repair* **4**, 22 (2011). 21970406 10.1186/1755-1536-4-22 PMC3204229

[39] Veidal, S. S. et al. MMP mediated degradation of type VI collagen is highly associated with liver fibrosis–identification and validation of a novel biochemical marker assay. *PLoS ONE* **6**, e24753 (2011). 21935455 10.1371/journal.pone.0024753 PMC3173456

[40] Bay-Jensen, A. C. et al. Circulating citrullinated vimentin fragments reflect disease burden in ankylosing spondylitis and have prognostic capacity for radiographic progression. *Arthritis Rheum.* **65**, 972–980 (2013). 23280360 10.1002/art.37843

[41] Leeming, D. J. et al. Enzyme-linked immunosorbent serum assays (ELISAs) for rat and human N-terminal pro-peptide of collagen type I (PINP)–assessment of corresponding epitopes. *Clin. Biochem.* **43**, 1249–1256 (2010). 20709044 10.1016/j.clinbiochem.2010.07.025

[42] Nielsen, M. J. et al. The neo-epitope specific PRO-C3 ELISA measures true formation of type III collagen associated with liver and muscle parameters. *Am. J. Transl. Res.* **5**, 303–315 (2013). 23634241 PMC3633973

[43] Leeming, D. J. et al. Enzyme-linked immunosorbent serum assay specific for the 7S domain of collagen type IV (P4NP 7S): A marker related to the extracellular matrix remodeling during liver fibrogenesis. *Hepatol. Res.* **42**, 482–493 (2012). 22221767 10.1111/j.1872-034X.2011.00946.x

[44] Frimodt-Møller, M. et al. A marker of type VI collagen formation (PRO-C6) is associated with higher arterial stiffness in type 1 diabetes. *Acta Diabetol.* **56**, 711–712 (2019). 30852680 10.1007/s00592-019-01306-9

[45] Chung, K. C. et al. Reliability and validity testing of the Michigan Hand Outcomes Questionnaire. *J. Hand Surg.* **23**, 575–587 (1998). 9708370 10.1016/S0363-5023(98)80042-7

[46] Gudmann, N. S. et al. Type I and III collagen turnover is increased in axial spondyloarthritis and psoriatic arthritis. Associations with disease activity and diagnostic capacity. *Clin. Exp. Rheumatol.* **35**, 653–659 (2017). 28240584

[47] Groen, S. S. et al. Op0031 serological collagen biomarkers can differentiate patients with psoriasis from psoriatic arthritis. *Ann. Rheum. Dis.* **81**(23), 2–23 (2022).

[48] Bay-Jensen, A. C. et al. Early changes in blood-based joint tissue destruction biomarkers are predictive of response to tocilizumab in the LITHE study. *Arthritis Res. Ther.* **18**, 7–15 (2016). 26787505 10.1186/s13075-015-0913-x PMC4719735

[49] Bay-Jensen, A. C. et al. Effect of tocilizumab combined with methotrexate on circulating biomarkers of synovium, cartilage, and bone in the LITHE study. *Semin. Arthritis Rheum.* **43**, 470–478 (2014). 23932312 10.1016/j.semarthrit.2013.07.008

[50] Hu, M. Y., Yang, Q. & Zheng, J. The association of psoriasis and hypertension: Focusing on anti-inflammatory therapies and immunological mechanisms. *Clin. Exp. Dermatol.* **45**, 836–40 (2020). 32789979 10.1111/ced.14327

