# Supplementary material for: Serum extracellular matrix biomarkers in rheumatoid arthritis, psoriatic arthritis and psoriasis and their association with hand function
Source: Sci Rep. 2025 Apr 21;15:13656. doi: 10.1038/s41598-025-98395-0 (PMC12009959; doi:10.1038/s41598-025-98395-0)
Supplement: Supplementary file 1 — Supplementary Information. [file 41598_2025_98395_MOESM1_ESM.docx]

Supplementary Table S1. Missing data points from hand function scores and biomarker measurements.

| **Variable** | **Missing data points (n)** |
| --- | --- |
| MHQ dominant | 14 |
| MHQ non dominant | 82 |
| Grip strength dominant hand | 3 |
| Grip strength non dominant hand | 14 |
| MPUT dominant hand | 75 |
| MPUT non dominant hand | 79 |
| ARG | 8 |
| C1M | 7 |
| C2M | 7 |
| C3M | 7 |
| C4M | 17 |
| C6M | 17 |
| PRO-C1 | 7 |
| PRO-C4 | 7 |
| VICM | 9 |
| PRO-C6 | 14 |
| PRO-C3 | 10 |

Abbreviations: MHQ, Michigan Hand Questionnaire; MPUT, Moberg Picking-Up Test; ARG, aggrecan ADAMTS degradation; C1M, MMP-2/9/13-degraded type I collagen; C2M, MMP (multiple) -degraded type II collagen; C3M, MMP-9-degraded type III collagen; C4M, MMP (multiple)-degraded type IV collagen; C6M, MMP (multiple)-degraded type IV collagen; PRO-C1, Type I collagen N-terminal propeptide; PRO-C4, Type IV 7S domain collagen; VICM, citrullinated and MMP-degraded vimentin; PRO-C6, Type VI collagen, alpha-3 chain, C5 domain; PRO-C3, type II collagen N-terminal propeptide.

Supplementary Table S2. Differences in hand tests between dominant and non-dominant hand for each of the groups.

|  | RA | | | PsA | | | PsO | | | Control | | | |
| --- | --- | --- | --- | --- | --- | --- | --- | --- | --- | --- | --- | --- | --- |
|  | D | ND | p-value | D | ND | p-value | D | ND | p-value | D | ND | p-value |  |
| Grip strength, pounds | 56.5 (32.6) | 59.0 (31.0) | 0.01 | 72.8 (35.0) | 72.7 (32.4) | 0.01 | 88.4 (36.4) | 80.3 (26.2) | 0.25 | 89.3 (25.3) | 91.2 (18.8) | 0.18 |  |
| MPUT, sec | 16.9 (7.3) | 20.3 (41.7) | 0.33 | 16.0 (7.7) | 17.0 (13.2) | 0.40 | 14.7 (5.0) | 14.5 (4.7) | 0.43 | 10.4 (2.1) | 10.8 (2.0) | 0.32 |  |
| MHQ, % | 63.9 (14.3) | 63.3 (9.7) | 0.47 | 69.2  (15.3) | 66.7 (9.2) | 0.43 | 75.5  (16.6) | 68.5 (10.4) | 0.50 | 88.6 (10.3) | 85.9  (12.2) | <0.01 |  |

Mean ± SD is presented. Kruskal-Wallis rank test was used. Abbreviations: RA, rheumatoid arthritis; PsA, psoriatic arthritis; PsO, psoriasis; D, dominant; ND, non-dominant; MPUT, Moberg Picking-Up Test; MHQ, Michigan Hand Questionnaire.

Supplementary Table S3. Comparison of linear regression models using robust standard errors with adjustments for age, sex and BMI (adjusted model) to those without adjustments (non-adjusted model). The reported data includes coefficient estimates along with their corresponding 95% confidence intervals for each model.

| Biomarker | contrast | Non-adjusted model | Adjusted model for age, sex and BMI |
| --- | --- | --- | --- |
| ARG | Control - PsA | -0.44[-0.54,-0.34] | 0.07[-0.04,0.18] |
|  | Control - PsO | -0.29[-0.36,-0.21] | 0.09[-0.02,0.2] |
|  | Control - RA | -0.57[-0.76,-0.39] | -0.03[-0.15,0.09] |
|  | PsA - RA | 0.14[0.06,0.22] | -0.1[-0.21,0.01] |
|  | PsO - PsA | -0.09[-0.17,-0.02] | -0.02[-0.11,0.07] |
|  | PsO - RA | -0.1[-0.17,-0.02] | -0.12[-0.23,-0.01] |
| C1M | Control - PsA | -0.09[-0.18,0] | -0.09[-0.19,0.01] |
|  | Control - PsO | 0.1[0,0.19] | -0.2[-0.29,-0.1] |
|  | Control - RA | -0.07[-0.17,0.02] | 0.06[-0.05,0.17] |
|  | PsA - RA | -0.08[-0.17,0.02] | 0.15[0.03,0.26] |
|  | PsO - PsA | -0.04[-0.13,0.05] | 0.11[0.01,0.22] |
|  | PsO - RA | 0.02[-0.05,0.1] | 0.26[0.14,0.37] |
| C2M | Control - PsA | -0.45[-0.55,-0.35] | -0.07[-0.17,0.03] |
|  | Control - PsO | -0.61[-0.78,-0.44] | -0.16[-0.26,-0.05] |
|  | Control - RA | -0.25[-0.33,-0.17] | 0.03[-0.08,0.14] |
|  | PsA - RA | -0.21[-0.3,-0.12] | 0.1[-0.01,0.22] |
|  | PsO - PsA | -0.12[-0.19,-0.04] | 0.08[-0.04,0.2] |
|  | PsO - RA | -0.12[-0.21,-0.04] | 0.19[0.06,0.32] |
| C3M | Control - PsA | -0.15[-0.26,-0.05] | -0.02[-0.12,0.08] |
|  | Control - PsO | 0.08[0.02,0.15] | -0.08[-0.17,0.02] |
|  | Control - RA | 0.07[0,0.13] | 0.12[0,0.24] |
|  | PsA - RA | 0.09[-0.01,0.19] | 0.15[0.02,0.27] |
|  | PsO - PsA | -0.09[-0.19,0] | 0.05[-0.04,0.14] |
|  | PsO - RA | 0.02[-0.07,0.11] | 0.2[0.08,0.32] |
| C4M | Control - PsA | -0.65[-0.8,-0.51] | -0.2[-0.28,-0.12] |
|  | Control - PsO | 0.25[0.18,0.32] | -0.21[-0.29,-0.12] |
|  | Control - RA | -0.4[-0.64,-0.17] | -0.07[-0.15,0.02] |
|  | PsA - RA | -0.13[-0.21,-0.04] | 0.14[0.05,0.22] |
|  | PsO - PsA | -0.1[-0.2,0] | 0[-0.08,0.09] |
|  | PsO - RA | 0.08[-0.03,0.2] | 0.14[0.05,0.23] |
| C6M | Control - PsA | 0.04[-0.05,0.14] | 0.17[0.09,0.26] |
|  | Control - PsO | 0.05[-0.02,0.13] | 0.13[0.06,0.2] |
|  | Control - RA | -0.01[-0.11,0.1] | 0.26[0.19,0.33] |
|  | PsA - RA | -0.01[-0.13,0.1] | 0.09[-0.01,0.19] |
|  | PsO - PsA | -0.04[-0.14,0.06] | 0.05[-0.05,0.14] |
|  | PsO - RA | -0.01[-0.12,0.09] | 0.13[0.04,0.23] |
| CRP | Control - PsA | 0.16[0.07,0.25] | -0.4[-0.6,-0.19] |
|  | Control - PsO | -0.21[-0.37,-0.06] | -0.46[-0.65,-0.28] |
|  | Control - RA | 0.16[0.03,0.28] | -0.24[-0.47,-0.02] |
|  | PsA - RA | 0.11[0.01,0.21] | 0.15[-0.07,0.37] |
|  | PsO - PsA | 0.13[0.02,0.24] | 0.07[-0.09,0.23] |
|  | PsO - RA | 0.17[-0.06,0.39] | 0.22[0,0.44] |
| PRO-C1 | Control - PsA | -0.11[-0.22,0] | -0.04[-0.13,0.05] |
|  | Control - PsO | 0.08[-0.03,0.19] | -0.09[-0.18,0.01] |
|  | Control - RA | 0.07[-0.05,0.19] | -0.04[-0.15,0.06] |
|  | PsA - RA | 0[-0.11,0.11] | -0.01[-0.12,0.1] |
|  | PsO - PsA | 0.03[-0.05,0.11] | 0.05[-0.06,0.16] |
|  | PsO - RA | -0.01[-0.1,0.08] | 0.04[-0.08,0.16] |
| PRO-C3 | Control - PsA | -0.11[-0.18,-0.04] | 0.05[-0.03,0.12] |
|  | Control - PsO | 0.12[0.01,0.23] | 0.08[0.01,0.14] |
|  | Control - RA | 0.06[-0.04,0.15] | 0.07[-0.02,0.15] |
|  | PsA - RA | 0.05[-0.05,0.15] | 0.02[-0.06,0.1] |
|  | PsO - PsA | -0.04[-0.12,0.05] | -0.03[-0.09,0.04] |
|  | PsO - RA | 0.08[-0.04,0.2] | -0.01[-0.09,0.06] |
| PRO-C4 | Control - PsA | 0.04[-0.12,0.2] | -0.04[-0.13,0.05] |
|  | Control - PsO | -0.04[-0.11,0.02] | -0.06[-0.14,0.02] |
|  | Control - RA | 0.01[-0.11,0.13] | 0.03[-0.09,0.14] |
|  | PsA - RA | 0.01[-0.08,0.1] | 0.07[-0.04,0.18] |
|  | PsO - PsA | 0.05[-0.06,0.16] | 0.02[-0.06,0.1] |
|  | PsO - RA | 0.02[-0.06,0.1] | 0.09[-0.02,0.2] |
| PRO-C6 | Control - PsA | 0.25[0.14,0.36] | -0.05[-0.12,0.03] |
|  | Control - PsO | 0.16[0.07,0.26] | 0.04[-0.04,0.13] |
|  | Control - RA | 0.21[0.09,0.33] | -0.07[-0.16,0.03] |
|  | PsA - RA | 0.12[0.03,0.21] | -0.02[-0.1,0.06] |
|  | PsO - PsA | -0.21[-0.36,-0.05] | -0.09[-0.16,-0.02] |
|  | PsO - RA | -0.12[-0.22,-0.03] | -0.11[-0.2,-0.02] |
| VICM | Control - PsA | 0.15[0.02,0.27] | -0.41[-0.51,-0.31] |
|  | Control - PsO | 0.21[-0.01,0.42] | -0.39[-0.49,-0.29] |
|  | Control - RA | 0.1[-0.01,0.21] | -0.64[-0.79,-0.5] |
|  | PsA - RA | -0.1[-0.21,0.01] | -0.23[-0.38,-0.08] |
|  | PsO - PsA | 0.05[-0.06,0.16] | -0.02[-0.14,0.09] |
|  | PsO - RA | -0.01[-0.09,0.06] | -0.25[-0.41,-0.09] |

Abbreviations PsA, psoriatic arthritis; : PsO, psoriasis; RA, rheumatoid arthritis; ARG, aggrecan ADAMTS degradation; C1M, MMP-2/9/13-degraded type I collagen; C2M, MMP (multiple) -degraded type II collagen; C3M, MMP-9-degraded type III collagen; C4M, MMP (multiple)-degraded type IV collagen; C6M, MMP (multiple)-degraded type IV collagen; PRO-C1, Type I collagen N-terminal propeptide; PRO-C4, Type IV 7S domain collagen; PRO-C3, type II collagen N-terminal propeptide; VICM, citrullinated and MMP-degraded vimentin.

Supplementary Figure S1. Unadjusted version of Figure 1, showing individual raw data points along with the mean ± standard deviation.


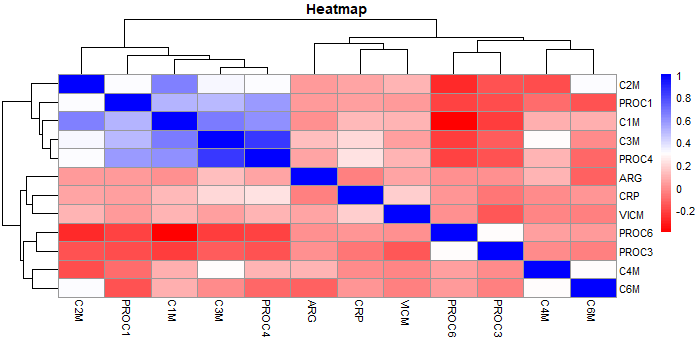


Supplementary Figure S2. This heatmap visualizes correlations between the 12 biomarkers studied, highlighting closely related biomarkers. The color scale represents positive correlations (blue) and negative correlations (red), with darker shades indicating stronger correlations. Abbreviations: ; C2M, MMP (multiple) -degraded type II collagen; PRO-C1, Type I collagen N-terminal propeptide; C1M, MMP-2/9/13-degraded type I collagen; C3M, MMP-9-degraded type III collagen; PRO-C4, Type IV 7S domain collagen; ARG, aggrecan ADAMTS degradation; CRP, C-reactive protein; VICM, citrullinated and MMP-degraded vimentin; PRO-C6, Type VI collagen, alpha-3 chain, C5 domain; PRO-C3, type II collagen N-terminal propeptide; C4M, MMP (multiple)-degraded type IV collagen; C6M, MMP (multiple)-degraded type IV collagen.
